# American Indian community engagement and the structural and social determinants of health: results from the THRIVE assessment

**DOI:** 10.3389/fpubh.2025.1608429

**Published:** 2025-08-22

**Authors:** Kellie Webb, Sadie Posey, Katherine Hirchak, Emily Beamon, Kelley Milligan, Sharon Wagon, Bethany Fatupaito, Allyson Kelley

**Affiliations:** ^1^Doya Natsu Healing Center, Eastern Shoshone Tribe, Fort Washakie, WY, United States; ^2^Allyson Kelley & Associates PLLC, Sisters, OR, United States; ^3^PRISM Collaborative, Department of Community and Behavioral Health, Elson S. Floyd College of Medicine, Washington State University, Spokane, WA, United States

**Keywords:** American Indian, social and structural determinants of health, validation, reservation community, equity

## Abstract

**Introduction:**

Engaging community members in the process of documenting health inequities is the first step in addressing public health challenges. This paper presents the community-driven adaptation process and results for the Tool for Health and Resilience in Vulnerable Environments (THRIVE) assessment, a social justice and equity-focused screening tool, in one reservation-based American Indian community in the US.

**Methods:**

Using principles of community-engaged research (CER) and community-based participatory research (CBPR), the authors describe the importance of co-creating data collection tools with community members to document the social and structural determinants of health. Authors describe a step-by-step approach to document inequities; this includes (1) recognizing the need to document inequities, (2) identifying existing tools to measure inequities, (3) adapting tools and piloting them with members of the community, (4) refining the tool based on community feedback, and (5) administering the tool to address needs identified.

**Results:**

Survey data from the THRIVE assessment and community-engaged process (*N* = 100) documented the social and structural determinants of health. The community-adapted THRIVE assessment examined the underlying factors of people, places, and opportunities that contribute to health inequities in the community. Results from this work show that the adapted THRIVE tool has high psychometric reliability (*α* = 0.957) and that community members feel strong about factors related to people and vulnerability, as well as factors related to place and equitable opportunities. The authors discuss future work and actions they will undertake to address community-identified concerns through community-based programs like complementary alternative medicine, harm reduction via mobile outreach, employment readiness and job training, sober housing, culturally centered treatment, and voucher-based programs to meet basic needs. The process presented underscores the importance of involving American Indian community members in co-creating data collection tools that represent the community’s needs and interests. This intersectional, holistic approach aims to enhance community conditions and achieve the highest possible health and well-being for American Indian communities.

## 1 Introduction

There are 9.7 million people of Indigenous peoples of Northern American descent (also referred to as American Indian and Alaska Native, AI/AN) living in the United States, Despite colonization, forced assimilation, removal from ancestral homelands, language and cultural erasure, and genocide, this group of individuals makes up approximately 3 % of the U.S. population (1). The voices of the AI/AN community have often been silenced through a number of tactics, including racism, discrimination, maintaining poverty levels in AI/AN communities, providing poor educational resources, and inadequate access to healthcare. As a result of these structural and social determinants of health, AI/AN often experience negative health outcomes like greater risk of mortality from suicide, consequences from drug and alcohol use, and shortened life expectancies (2).

The federal government’s actions to oppress, suppress, and undercount AI/ANs have been perpetuated through issues with misclassifications of race/ethnicity, ancestry, and tribal affiliation among survey and census-level data (3). Policymakers have alerted that AI/AN communities are historically underrepresented and undercounted in federal surveys like the national U.S. Census (4). AI/AN populations are often undercounted, misclassified, and inconsistently measured, impacting subsequent estimates of AI/AN health concern (5). When AI/AN health concerns are underestimated, funding opportunities, priorities of policy makers, and the development of AI/AN-specific scientific research are negatively impacted.

Involving community members in designing and administering data collection instruments can lead to improved data quality, increased capacity at the community level, and partnerships with communities. Community-based researchers often utilize community-engaged research (CER) (6), community-based participatory research approach (CBPR) (7), or a Tribal participatory approach (8) to design data collection instruments and research protocols. Engaged and participatory approaches are often used by public health professionals working in AI/AN community settings to conduct public health practice work- including the development and validation of community surveys (9). This process would include key AI/AN personnel, it would begin with Tribal involvement regarding documentation of the main problem/challenge, and would initiate the creation of a plan to address the problem (10).

The Structural and Social Determinants of Health (SSDOH) are critical to document when addressing health disparities and inequities in AI/AN populations (11, 12). The Indian Health Service and the Department of Health and Human Services call attention to the need for SSDOH integration and collaborative strategies to address inequities (13). Similarly, the United States Department of Health and Human Services prioritizes SSDOH through an Action Plan that focuses on three main goals: (1) building a robust and interconnected data infrastructure, (2) improving access to and affordability of equitably delivered healthcare services, and (3) adopting whole-government approaches to enhance population health and wellbeing. The World Health Organization (WHO) even calls for prioritizing the Indigenous Determinants of Health. The WHO explicitly recognizes Indigeneity and colonialism as overarching determinants of health, providing direct support for Indigenous people and communities, and implementing plans that are strength-based and culturally grounded (14). Given these priorities, however, there has been minimal progress in documenting the SSDOH from an AI/AN community-engaged perspective.

When considering structural and SSDOH factors, along with health disparities and inequities in AI/AN communities, it is essential to recognize the role of intersectionality. As demonstrated by other researchers, discrimination, racism, poverty, and homelessness are risk factors that are multiplicative in their effect on substance use, addiction, and mental health disorders (15). For various reasons, there is an intersectionality among the determinants of risk factors, the risk factors themselves, and the diseases caused by these risk factors (16). The intersectionality of any given health disparity in a community often includes income, employment, education, healthcare access, cultural connections, and historical trauma (17). Intersectionality aligns with an Indigenous community framework, where all living beings are interconnected and in relationship with one another. The Medicine Wheel symbol (mental, physical, emotional, spiritual) is one of the first examples of intersectionality in the published literature and continues to be used as a method for collaborative evaluation and decision-making with AI/AN peoples (18–20). The Medicine Wheel involves a circle teaching and values, that all growing things grow in a system of circles and cycles, there are four quadrants of the Medicine Wheel that represent different cycles of life and directions. Tribes have different uses and teachings around the Medicine Wheel. In one example, spiritual health is often placed in the north, the time of life is elder, and the season is winter. Emotional health is the direction of the east, the time of life is baby, and the season is spring. Physical health is the direction of the south. The time of life is youth, and the season is summer. Mental health is the west, the time of life is adult, and the season is fall.

While there are definitions of what SSDOH are among AI/AN groups, there are no best practices for actually measuring SSDOH in these populations. Stemming from colonial, historical, and present-day structural inequities, structural determinants of SSDOH include public policies, social policies, and governance systems. Structural inequities have been defined as the systematic disadvantage of one social group over another (21). Within a public health perspective, it is also classified as the inequitable distribution of power, money, and resources that influence health and safety outcomes and shape where people are born, live, work, grow, and age (22).

Efforts to screen individuals and document SSDOH factors in clinical settings with non-AI/ANs have been studied (23). Findings from these SSDOH-focused assessments have informed programs, elevated policies, and monitored the progress of public health equity-focused interventions. Public health programs and universities have promoted tools like the Tool for Health and Resilience in Vulnerable Environments (THRIVE) to document SSDOH perspectives and improve health equity by reducing disparate health outcomes (24). The THRIVE assessment has been utilized among non-AI/AN populations to identify and prioritize factors that can serve as the basis for local action planning and health equity. THRIVE challenges communities to think about health and safety, and the value of community and cultural resilience. THRIVE is an evidence-informed, practiced based framework for change and supports communities as they find solutions to their challenges that reflect community values and cultural norms (25). THRIVE also builds community strengths and relies on local community leadership—these factors made THRIVE the best decision for the Healing Center to measure SSDOH.

For the purposes of this study, we reviewed the original THRIVE assessment developed by the Prevention Institute (24). We adapted it using published best practices for adapting and validating scales with Indigenous community involvement (9). The first adapted THRIVE assessment included the Prevention Institute’s framework, using 11 questions, and three categories related to community effectiveness ratings, priority ratings, and top picks (see Supplemental material 1). In contrast, this validation process of adapting an SSDOH screening tool and establishing its psychometric properties with AI/AN reservation communities has not been studied.

This paper presents a community-engaged process of adapting the THRIVE assessment tool with one AI/AN community to document community conditions that influence health and drive inequities. The community-adapted THRIVE assessment focuses on place, equitable opportunities, people, and self-governance/healthcare.

## 2 Materials and methods

### 2.1 Ethics statement

The Tribe is a sovereign nation that approves and reviews all data collection instruments and plans before they are used within their community. The Healing Center Director met with the Tribal Council and presented the initial THRIVE Assessment tool for them to review. The Tribal Council did not have additional questions or concerns regarding the THRIVE Assessment. Tribal Council approved this instrument and granted permission to collect information from its community members during an initial pilot period. The Director returned to the Tribal Council and presented results from the initial data collection and the final adapted THRIVE Assessment tool. The Tribal Council approved the updated tool for community data collection.

### 2.2 Adapting the THRIVE assessment

Consistent with a CBPR approach, our evaluation team met weekly with the Healing Center staff and community members to review the original THRIVE assessment. Together we designed the THRIVE Community Assessment Worksheet. This was similar to the original Prevention Institute THRIVE Assessment but included 11 SSDOH related questions and two demographic questions. We also used the Healing Center’s style guide for recognition and cultural relevance. We added a question, “Where do you live?” since the original THRIVE Assessment did not include this question (see Supplemental material). Over the course of 2 months, we piloted the adapted THRIVE Assessment with seven community members attending an AI/AN reservation-based Healing Center events. Feedback from community members indicated that the survey was too long, the scoring was confusing, the priority ratings lacked cultural relevance, and it was not possible to select just three options. The language used to describe each THRIVE Factor was lengthy and difficult to understand.

Table 1 outlines the original THRIVE Assessment and AI/AN adaptations made for the community based on pilot feedback. We worked with the Healing Center’s Communication Staff to post the adapted THRIVE Assessment online survey on social media platforms, including Facebook and the Healing Center’s website. We offered $20 gift cards for paper surveys completed at the Healing Center to encourage participation. Unfortunately, online surveys were erroneously completed by bots, resulting in 7,766 surveys from multiple states, countries, and locations beyond the reservation. Due to the infiltration of bots into the online surveys, none of these responses were used in the analysis of the adapted THRIVE assessment. The Director presented this information to the Tribal Council and received approval to modify the survey using paper methods to be completed in person. This new in-person data collection process addressed the issue with bots and ensured that the survey data represented individuals in the community.

**Table 1:** Original and adapted THRIVE assessment.

Original THRIVE assessment		Adapted THRIVE assessment	
Cluster	THRIVE factor	Cluster	THRIVE Factor
People–Score, rating, Top 3, Write-in	Social networks Act for the common good Norms and culture Write in	People–5 Point Likert Scale (Crisis to Thriving)	Family, relationships, trust Act for the common good Norms and culture uplift wellness, values, traditions
Place–Score, rating, Top 3, Write-in	What sold and promoted Look, feel, safety Parks and open spaces Getting around Housing Air, water, and soil Arts and cultural expression Write-in	Place–5 Point Likert Scale (Crisis to Thriving)	Safe, healthy, affordable products/services Safe surroundings Parks and open spaces Getting around Housing Air, water, soil safe Positive cultural values expressed
Equitable Opportunity–Score, rating, Top 3, Write-in	Living wages and wealth Education Write-in	Equitable Opportunity–5 Point Likert Scale (Crisis to Thriving)	Employment pays a living wage, investments, Local ownership of houses, land, assets Education is high quality, serves all
N/A	N/A	Self-Governance–5 Point Likert Scale (Crisis to Thriving)	The current healthcare system is high quality
N/A	N/A	Crisis–Open text	What would you or a loved one do in crisis?

We used this community feedback to create a modified survey. The new THRIVE assessment included 14 items and was simplified to include a 5-point Likert-type rating scale (*1 = Crisis, 2 = Vulnerable, 3 = Safe, 4 = Stable, 5 = Thriving*) for five THRIVE factors- Place, People, Equal Opportunity, and Tribal Self-Governance (see Supplemental material). Place questions relate to safety, surroundings, parks, housing, environment, cultural values, and transportation. People questions include norms, culture, family, and values. Equal opportunity focused on local ownership of houses and land, education quality, and employment opportunities. Tribal Self-Governance was added to the THRIVE Assessment because the Healing Center and Tribal Council are in the process of changing healthcare systems and delivery and wanted feedback regarding healthcare. Instructions were: Read each statement. How well is your community doing? Give your community a score of 1 to 5. For example, for the THRIVE factor People, a statement was, “People act for the common good” (see Supplemental material 1).

We added three questions to the THRIVE Assessment regarding insurance and healthcare services. One additional question asked, “What would you do if you or a loved one experienced a crisis on the Wind River Reservation?” with fixed responses and the option to select all responses that apply. The last question asked, “Please provide any additional comments about your community in the space below.” Data were collected using the online surveying platform- Qualtrics and exported as a paper survey for individuals with limited access to the Internet.

### 2.3 Data collection and analyses

The evaluation team worked with the Healing Center to collect printed THRIVE surveys at community outreach events, after groups, talking circles, and other opportunities from November 2023 to November 2024. Most outreach occurred via word of mouth and at local Tribal events. To participate in the survey, we required participants to be living in a community located on or near the reservation, aged 18 or older, and willing to complete the paper survey. Upon completion, participants each received a $20 gift card. The Director scanned all paper copies of surveys to the evaluation team. A Tribal community evaluation team member was responsible for data entry and cleaning.

Descriptive statistics were performed for all 14 THRIVE items (see Table 2). Internal consistency was estimated for each applicable factor using Cronbach’s alpha (*α*). All analyses were performed in SPSS v26.

**Table 2:** Structural determinants of health ratings.

Item	Crisis	Vulnerable	Safe	Stable	Thriving	Total *N*
	*N* (%)	*N* (%)	*N* (%)	*N* (%)	*N* (%)	
People Subscale (3)						
Family, relationships, and trust	11 (11)	22 (22)	21 (21)	34 (34)	11 (11)	99
People act for the common good	10 (10)	26 (26)	29 (29)	28 (28)	6 (6)	99
Norms and culture uplift wellness values and traditions	7 (7)	18 (18)	31 (32)	27 (28)	15 (15)	98
Place Subscale (7)						
Safe, healthy and affordable products and services	9 (9)	32 (32)	24 (24)	24 (24)	11 (11)	100
Surroundings are safe, well maintained for all people	14 (15)	23 (25)	25 (26)	20 (21)	12 (13)	94
Parks and open spaces are accessible, safe, clean and appeal to multiple generations	18 (18)	32 (32)	23 (23)	14 (14)	12 (12)	99
Getting around is safe, reliable and affordable for everyone	16 (17)	32 (33)	12 (13)	25 (26)	11 (11)	96
Housing is high quality, affordable, and available to mixed income levels	26 (27)	27 (28)	17 (17)	19 (19)	9 (9)	98
Air, water, and soil are safe and nontoxic	12 (12)	35 (35)	25 (25)	18 (18)	10 (10)	100
Positive cultural values expressed in arts and what is seen and felt on the reservation	12 (12)	29 (29)	23 (23)	23 (23)	13 (13)	100
Equal Opportunity Subscale (3)						
Employment pays a living wage or salary; there are investment opportunities	17 (17)	34 (35)	25 (26)	17 (17)	5 (5)	98
Local ownership of houses, land and other assets is common	18 (19)	33 (35)	22 (23)	16 (17)	6 (6)	95
Education is high quality and serves all learners	16 (16)	31 (32)	20 (21)	20 (21)	10 (10)	97
Tribal Self-Governance (1)						
Current healthcare is high quality	15 (15)	34 (34)	22 (22)	21 (21)	7 (7)	99

## 3 Results

A total of 100 participants completed the adapted THRIVE surveys, representing seven separate communities on or bordering the reservation. Participant characteristics can be found in Table 3. Nearly, 56% of participants were categorized as 41 years of age and above. Of the respondents who completed the survey, 53% reported having some form of insurance, with 61% indicating they receive healthcare services at the Indian Health Service. We also asked participants to indicate what they would do in a crisis situation by selecting all applicable responses. When in crisis, participants most often endorsed that they would “reach out to a family member or friend,” “contact a local program in place to address the crisis,” or “seek support from a pastor or a spiritual advisor.”

**Table 3:** THRIVE respondent characteristics (*N* = 100).

Variable		*N* (%)
Age		
	0–20	6 (6)
	21–30	14 (14)
	31–40	25 (25)
	41–50	21 (21)
	50+	35 (34)
Location		
	Community 1	29 (28)
	Community 2	33 (32)
	Community 3	1 (1)
	Community 5	10 (10)
	Community 6	8 (8)
	Community 7	8 (8)
	Community 8	13 (13)
Insurance Coverage		
	Yes	53 (53)
	No	47 (47)
Insurance Type		
	Medicaid	29 (21)
	Kidcare	4 (3)
	Medicare	13 (9)
	Veterans Benefits	7 (5)
	Private Insurance	4 (3)
	Employer paid insurance	33 (24)
	Indian Health Insurance Only	40 (29)
	None	11 (8)
	Other	2 (1)
Where do you access healthcare services?		
	IHS	75 (61)
	Veteran Affairs	9 (7)
	Fremont Public Health	5 (4)
	Wyoming State Health Dep	4 (3)
	Private Medical Provider	8 (7)
	Nowhere	6 (5)
	Other	12 (10)
What would you do in a crisis?		
	Call or text the 988 Lifeline	21 (15)
	Reach out to a family member or a friend	62 (44)
	Seek support from a pastor or a spiritual advisor	26 (19)
	Contact a local program in place to address the crisis (recovery, behavioral health, law enforcement, social services)	30 (21)
	Other (local and state medical providers / healthcare services)	1 (1)

### 3.1 Descriptive and inferential analyses

Measures of SSDOH were evaluated for their psychometric properties and utility. Cronbach’s Alphas for the scale were acceptable for all constructs measured (n = 14 items, *α* = 0.957). Scale items, item-total correlations, and Alphas (denoted by α) are described in Table 2.

Validity among variables was assessed by grouping items that measured the same constructs (Table 4).

**Table 4:** Adapted THRIVE scale descriptives and reliability scores for constructs.

Construct	Mean	Standard Deviation	Cronbach’s Alpha
People (3 items)	3.10	0.99	0.831
Place (7 items)	2.81	1.05	0.935
Equal Opportunity (3 items)	2.63	1.06	0.878
Tribal Self-Governance Healthcare (1 item)	2.71	1.17	N/A

We reviewed the total THRIVE Assessment scores based on the 5-point Likert Scale to determine if there were any differences by age group or location, but none were found. Similarly, there was insufficient variability to check for differences in healthcare services between groups. There was no difference between those with insurance and those without insurance across THRIVE scores.

## 4 Discussion

This is the first pilot AI/AN community survey to adapt and establish the reliability of an SSDOH screening tool, as well as assess the structural and SSDOH in an AI/AN reservation community. Results from the adapted THRIVE assessment indicate that people, place, and equitable opportunity remain crucial factors in addressing health disparities among AI/AN populations. Specifically, SSDOH related to People statements were the highest among respondents (rated as stable to thriving), where the items “People - Family, relationships, and trust” were rated the highest among respondents, followed by “People - Norms, culture, uplift wellness values and traditions.” This finding is consistent with previous research in the community, which highlights the importance of family, kinship systems, and community as vital components of well-being and generational healing (26). However, Place statements ranked lower. Most community members considered their community’s safety, transportation, housing, environment, and cultural values as vulnerable. The Healing Center recognizes the need to address these conditions and utilizes a mobile outreach van to better serve higher-need communities (27). Concerning equal opportunity, this condition was also vulnerable, as employment, housing ownership, and education need to be improved. The last statement relating to self-governance and health was also vulnerable. This was essential information, as the Tribe is in the process of self-governing all healthcare services on the reservation, and community perspectives on their governance are crucial to the implementation of these services. Previous researchers have highlighted the well-documented disparities in health outcomes and mortality among AI/AN populations, which are attributed to systemic differences in healthcare and housing access (28). Our findings are similar, where Place statements and housing were rated vulnerable. Housing and place-based vulnerability ratings could be linked to the historical context of forced removal from land, housing, and the Indian Removal Act of 1830 and the Indian Relocation Act of 1956. The lingering impacts of these genocidal acts have reverberating impacts on the health, safety, and access AI/AN people have with their land and their homes (29). However, the strength of family, kinship systems and community support is evidenced in who people will turn to in a crisis. Most participants would go to a family or friend first, followed by a local program to address crisis. This is consistent with previous research in other AI/AN populations that found strong family ties, and social cohesion during times of crisis is a common AI/AN response (30).

## 5 Limitations and future directions

Our findings demonstrate the process of engaging AI/AN communities in survey design and data collection. A strength of this approach is that, through true partnership and engagement, we adapted and established the psychometric properties of the THRIVE survey to assess the structural and SSDOH in AI/AN communities. There are a few limitations. First, the sample size is relatively small, and data was collected using convenience sampling methods. In one community, there was just one representative and, in another community, there were no representatives. This limits the generalizability of these findings to this reservation community and other nations. Notably, there are 574 federally recognized sovereign AI/AN nations with unique customs, language, histories, and teachings. Due to the heterogeneity across all AI/AN populations in the United States, this THRIVE assessment may not be appropriate for every Tribal nation, Indigenous community, or First Nation population. Second, there were no differences in total scores based on age group, location, insurance, or healthcare status. This could be viewed as a strength because tribal identity rather than demographic characteristics are defining community response to THRIVE factors. As more data are collected using the adapted THRIVE assessment, differences in structural and SSDOH perspectives may emerge. Finally, there are differences in how healthcare coverage and insurance are defined in the literature and in surveys like this. Most respondents accessed healthcare services at the Indian Health Service, and this is not insurance, but rather a treaty right to AI/ANs. Data regarding insurance coverage and status should be interpreted with caution.

This community-engaged process highlights the need for ongoing advocacy, funding, and policy changes to support Tribal communities and programs adequately, utilizing structural and social determinants of health (SSDOH) as a guide (31). These preliminary findings require additional validation with other tribal communities and in other parts of the United States to fully understand the use and power of SSDH focused tools like THRIVE to elevate equity and advocacy. The Healing Center plans to continue collecting data using the validated THRIVE assessment and share ongoing results with Tribal leaders, funding agencies, and programs to address structural and SSDOH factors that impact the health and wellbeing of AI/AN community members. To address some of the needs identified in the THRIVE assessment, the tribe will continue to fund and implement community-based programs and services like complementary alternative medicine, harm reduction via mobile outreach (i.e., including a range of services that work to decrease stigma, including providing clothes, food and other necessities addressing the SSDOH), employment readiness and job training, sober housing, culturally centered treatment, and voucher-based programs to meet basic needs. This intersectional, holistic, and community-based approach will enhance community conditions to ensure AI/AN individuals achieve the highest possible health and well-being. The Healing Center’s message is that every journey begins with one step. The journey of documenting the structural and SSDOH using the THRIVE assessment is a first step in ensuring improved health and wellbeing for future generations, as one Lakota phrase reminds us, “So that the people may live.”

## Data Availability

The original contributions presented in the study are included in the article/Supplementary material, further inquiries can be directed to the corresponding author.
