# Supplementary material for: American Indian community engagement and the structural and social determinants of health: results from the THRIVE assessment
Source: Front Public Health. 2025 Aug 22;13:1608429. doi: 10.3389/fpubh.2025.1608429 (PMC12411181; doi:10.3389/fpubh.2025.1608429)
Supplement: Supplementary file 1 [file Data_Sheet_1.pdf]

EASTERN SHOSHONE RECOVERY
every journey begins with just one step

THRIVE COMMUNITY ASSESSMENT WORKSHEET

This assessment helps to identify and prioritize the THRIVE (Tool for Health & Resilience in Vulnerable Environments) factors that can be the basis for a local action plan to improve health equity. The goal of health equity is the reduction of disparate health outcomes that are unnecessary, avoidable, and unjust and that typically are the result of social and historical bias. As a determinant of health and safety, each factor in this assessment can help to ensure that all communities have access to the same opportunities for good health and a fulfilling and productive life.

ESR plans to use this information to plan future programs, inform policy, and monitor changes in community conditions over time.

WORKSHEET INSTRUCTIONS:

Complete steps 1-3 to identify the opportunities to improve health and safety and decrease health inequities in your community. The tool enables you to rate factors in the social-cultural environment (people), physical environment (place), and economic environment (equitable opportunity).

Step 1:
COMMUNITY EFFECTIVENESS SCORE

(A – F): How well is your community doing on this factor? Using a scale of A (excellent) to F (failing), rate how well this factor is currently being addressed in your community. Please bubble in your response.

Step 2:
PRIORITY RATING

What are your priorities for increasing health and safety? Given what you know about the effectiveness of current efforts to address each of the factors, how would you rate the priority of future efforts to increase health and decrease inequity for each factor? Please bubble in your choices.

Step 3:
TOP THREE PRIORITIES

What should your community focus on first? Based on your effectiveness and priority ratings, bubble in the three areas across all twelve factors that you feel are most important to address in your community with the goal of increasing health and safety and reducing health inequities.

Adapted from the Prevention Institute THRIVE project, available at: www.preventioninstitute.org

DEMOGRAPHIC INFORMATION (OPTIONAL):

Age:	Where do you live?	
☐ 0- 17	☐ Fort Washakie	☐ St. Stephens
☐ 18-35	☐ Riverton	☐ Crow Heart
☐ 36-64	☐ Lander	☐ Other: ______________
☐ 64+	☐ Ethete	
	☐ Arapaho	

RACIAL AND JUSTICE

Cluster	THRIVE Factor	Community Effectivenes Score<br>A B C D E F	Priority Rating<br>Low - Med -High	Top 3 Picks
People	1. **Social Networks & Trust:** Trusting relationships among community members built upon a shared history, mutual obligations, opportunities to exchange information, and that foster the formation of new, and strengthen existing, connections.	A	Low	☐
	2. **Participation & Willingness to Act for the Common Good:** Individual capacity, desire, and ability to participate, communicate, and work to improve the community; meaningful participation by tribal leadership; involvement in the community such as through local community and social organizations and participation in the political, advocacy, or tribal resolution process.	A	Low	☐
	3. **Norms and Culture:** Broadly accepted behaviors to which people generally conform that promote health, wellness and safety among all community tribal members; discourage behaviors that inflict emotional or physical distress on others; and reward behaviors that positively affect others; Norms include values and practices stemming from spiritual and cultural belief systems that are often linked to those core personal and community characteristics from which identity derives.	A	Low	☐
	Write-in at the People Level:	A	Low	☐

Adapted from the Prevention Institute THRIVE project, available at: www.preventioninstitute.org

Cluster	THRIVE Factor	Community Effectivenes Score<br>A B C D E F	Priority Rating<br>Low - Med -High	Top 3 Picks
Place	4. **What's Sold & How It's Promoted:** Availability and promotion of safe, healthy, affordable, culturally appropriate products and services (e.g. food, pharmacies, books and school supplies, sports equipment, arts and crafts supplies, and other recreational items); and the limited promotion, availability, and concentration of potentially harmful products and services (e.g. tobacco, firearms, alcohol, and other drugs).	A	Low	☐
	5. **Look, Feel & Safety:** Surroundings that are well-maintained, appealing, perceived to be safe, and culturally inviting for all residents.	A	Low	☐
	6. **Parks and Open Spaces:** Availability and access to safe, clean parks, green space, and open areas that appeal to interests and activities across the generations.	A	Low	☐
	7. **Getting Around:** Availability of safe, reliable, accessible and affordable ways for people to move around, including public transit, walking, biking, and using devices that aid mobility.	A	Low	☐
	8. **Housing:** High-quality, safe and affordable housing that is accessible for residents with mixed income levels.	A	Low	☐
	9. **Air, Water & Soil:** Safe and non-toxic water, soil, indoor and outdoor air.	A	Low	☐
	10. **Arts & Cultural Expression:** Abundant opportunities exist within the community for cultural and artistic expression and participation, and for positive cultural values to be expressed through the arts; and arts and culture positively reflect and value the backgrounds of all community residents.	A	Low	☐
	**Write-in at the Place Level:**	A	Low	☐

Adapted from the Prevention Institute THRIVE project, available at: www.preventioninstitute.org

Cluster	THRIVE Factor	Community Effectivenes Score<br>A B C D E F	Priority Rating<br>Low - Med -High	Top 3 Picks
Equity Opportunity	11. **Living Wages and Local Wealth:** Local ownership of houses, land, and other assets; accessible local employment that pays living wages and salaries; and access to investment opportunities.	A	Low	☐
	11. **Education:** High-quality education and literacy development for all ages that effectively serves all learners.	A	Low	☐

For more information about this assessment, conTact:

Kellie Webb
ESR Director
esr.director@gmail.com

Adapted from the Prevention Institute THRIVE project, available at: www.preventioninstitute.org
