# Supplementary material for: American Indian community engagement and the structural and social determinants of health: results from the THRIVE assessment
Source: Front Public Health. 2025 Aug 22;13:1608429. doi: 10.3389/fpubh.2025.1608429 (PMC12411181; doi:10.3389/fpubh.2025.1608429)
Supplement: Supplementary file 2 [file Data_Sheet_2.docx]

THRIVE Assessment October 2023

Start of Block: Default Question Block

Q1 This assessment will help the Healing Center understand more about the health, resilience, and environment on the Reservation. We do not want you to include your name or any identifying information; you do not have to complete this if you do not want to.

What is your age? 

- 0 to 20
- 21 to 30
- 31 to 40
- 41 to 50
- 50 and older

Q2 Where do you live?

- Community names redacted
- Other, please describe __________________________________________________

Q3 Read the statements below. How well is your community doing? Give your community a score of 1 to 5. 1-Crisis, 2- Vulnerable, 3- Safe, 4- Stable, 5- Thriving. Write a number between 1 and 5 on the line next to each statement.

|  | Crisis | Vulnerable | Safe | Stable | Thriving |
| --- | --- | --- | --- | --- | --- |
| People- Family, relationships, and trust |  |  |  |  |  |
| People-People act for the common good |  |  |  |  |  |
| People Norms and culture uplift wellness values, and traditions |  |  |  |  |  |
| Place- Safe, healthy and affordable products and services |  |  |  |  |  |
| Place- Surroundings are safe, well maintained for all people |  |  |  |  |  |
| Place- Parks and open spaces are accessible, safe, clean and appeal to multiple generations |  |  |  |  |  |
| Place- Getting around is safe, reliable and affordable for everyone |  |  |  |  |  |
| Place-Housing is high quality, affordable, and available to mixed income levels |  |  |  |  |  |
| Place-Air, water, and soil are safe and nontoxic |  |  |  |  |  |
| Place- Positive cultural values expressed in arts and what is seen and felt on the reservation |  |  |  |  |  |
| Equal opportunity- Employment pays a living wage or salary, there are investment opportunities |  |  |  |  |  |
| Equal opportunity-Local ownership of houses, land and other assets is common |  |  |  |  |  |
| Equal opportunity- Education is high quality and serves all learners |  |  |  |  |  |
| Tribal Self-Governance- Current healthcare is high quality |  |  |  |  |  |

Q4 What would you do if you or a loved one experienced a crisis on the Wind River Reservation? Select all that apply.

- Call or text the 988 Lifeline
- Reach out to a family member or a Friend
- Seek support from a pastor or a spiritual advisor
- Contact a local program in place to address the crisis (recovery, behavioral health, law enforcement, social services)
- Other, please describe __________________________________________________

Q5 Do you have health insurance?

- Yes
- No

Display This Question:

If Do you have health insurance? = Yes

Q6 If you do have insurance, which coverage do you have? Select all that apply.

- Medicaid
- Kidcare
- Medicare
- Veterans Benefits
- Private Insurance
- Employer-paid insurance
- Indian Health Service Only
- None

Q7 Where do you get healthcare services? Select all that apply.

- Indian Health Service
- Veteran Affairs
- Fremont Public Health
- Wyoming State Health Department
- Private Medical Provider
- No where
- Other:________________________ __________________________________________________

Q8 Please provide any additional comments about your community in the space below.

________________________________________________________________End of Block: Default Question Block
