# Supplementary figures and images for: American Indian community engagement and the structural and social determinants of health: results from the THRIVE assessment
Source: Front Public Health. 2025 Aug 22;13:1608429. doi: 10.3389/fpubh.2025.1608429 (PMC12411181; doi:10.3389/fpubh.2025.1608429)

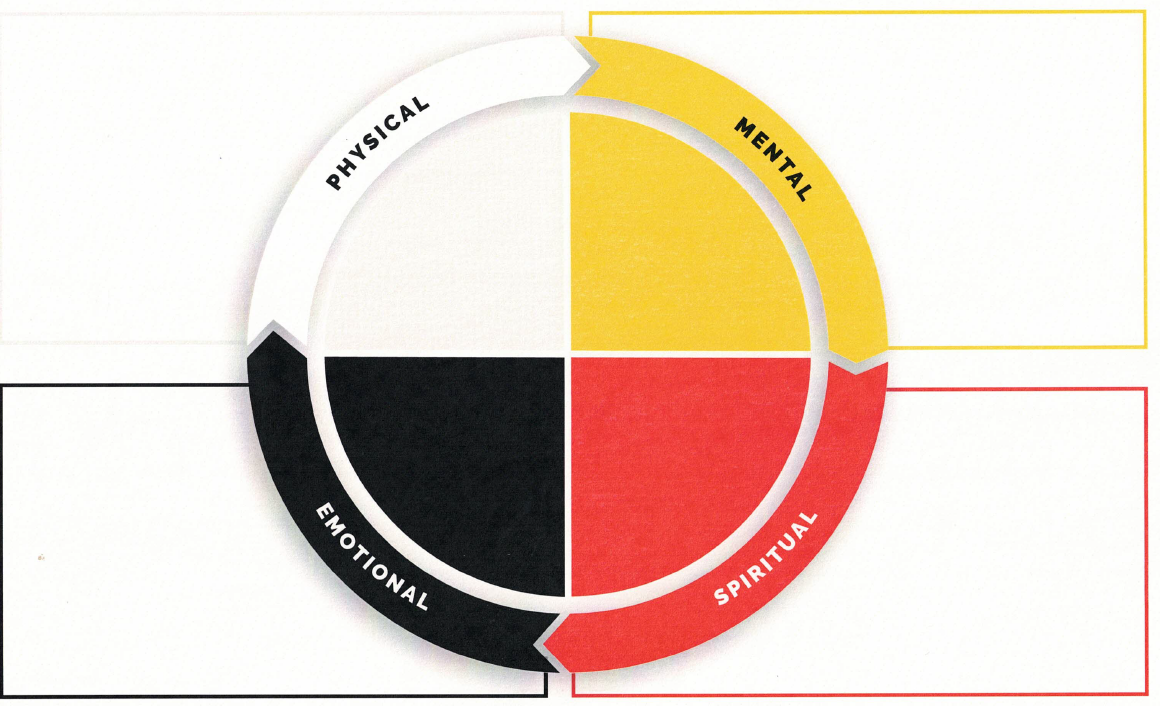

Supplement: Supplementary file 3 [file Image_1.png]

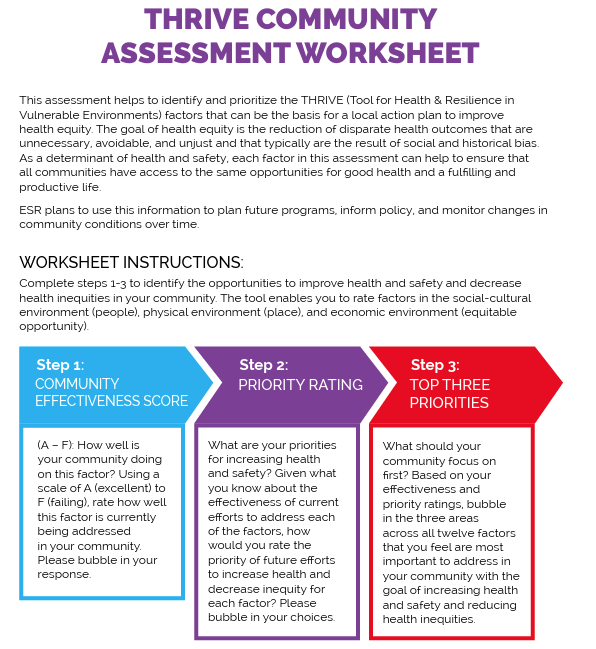

Supplement: Supplementary file 4 [file Image_2.png]
